# Diagnostic and Therapeutic Potential of Circulating-Free DNA and Cell-Free RNA in Cancer Management

**DOI:** 10.3390/biomedicines10082047

**Published:** 2022-08-22

**Authors:** Sadia Hassan, Adeeb Shehzad, Shahid Ali Khan, Waheed Miran, Salman Khan, Young-Sup Lee

**Affiliations:** 1 Department of Biomedical Engineering and Sciences, School of Mechanical and Manufacturing Engineering (SMME), National University of Sciences and Technology (NUST), Islamabad 44000, Pakistan; 2 Department of Chemistry, School of Natural Sciences (SNS), National University of Sciences and Technology (NUST), Islamabad 44000, Pakistan; 3 Department of Chemical Engineering, School of Chemical and Materials Engineering National University of Sciences and Technology (NUST), Islamabad 44000, Pakistan; 4 Department of pharmacy, Quaid-i-Azam University, Islamabad 44000, Pakistan; 5 School of Life Sciences, College of Natural Sciences, Kyungpook National University, Daegu 41566, Korea

**Keywords:** liquid biopsy, cancer, anticancer therapy, diagnosis, *cf*RNA, *cf*DNA, biomarkers

## Abstract

Over time, molecular biology and genomics techniques have been developed to speed up the early diagnosis and clinical management of cancer. These therapies are often most effective when administered to the subset of malignancies harboring the target identified by molecular testing. Important advances in applying molecular testing involve circulating-free DNA (*cf*DNA)- and cell-free RNA (*cf*RNA)-based liquid biopsies for the diagnosis, prognosis, prediction, and treatment of cancer. Both *cf*DNA and *cf*RNA are sensitive and specific biomarkers for cancer detection, which have been clinically proven through multiple randomized and prospective trials. These help in cancer management based on the noninvasive evaluation of size, quantity, and point mutations, as well as copy number alterations at the tumor site. Moreover, personalized detection of *ct*DNA helps in adjuvant therapeutics and predicts the chances of recurrence of cancer and resistance to cancer therapy. Despite the controversial diagnostic values of *cf*DNA and *cf*RNA, many clinical trials have been completed, and the Food and Drug Administration has approved many multigene assays to detect genetic alterations in the *cf*DNA of cancer patients. In this review, we underpin the recent advances in the physiological roles of *cf*DNA and *cf*RNA, as well as their roles in cancer detection by highlighting recent clinical trials and their roles as prognostic and predictive markers in cancer management.

## 1. Introduction

Cancer is a complex and multifactorial disease with debilitating effects, orchestrated by a plethora of genetic and environmental factors and associated with serious comorbidities. As cancer is one of the leading causes of death after cardiovascular diseases [1], many researchers have spent their time, efforts, and resources on finding new cancer management solutions to reduce the mortality rate; ergo, the cancer death rate decreased by 32% between 1991 and 2019. Many efforts are focused on the early diagnosis and prediction of reoccurrence of disease; for that purpose, tissue biopsies and imaging-based technologies have been introduced. Liquid biopsies (mainly cell-free DNA and RNA) are more desirable and attractive options due to their non-invasiveness, easiness, and effectiveness.

Cell-free DNA (*cf*DNA) and cell-free RNA (*cf*RNA) are, respectively, fragmented DNA and RNA that move freely in body fluids (rather than being encapsulated inside the cells). *cf*DNA gained global recognition in the 1960s when it was hypothesized that it is somehow related to the metastasis of cancer; however, it was first reported in human blood by Mandel and Metais in 1948 [2]. In 1965, the relationship between cancer and *cf*DNA was hypothesized [3]; a year later, high levels of *cf*DNA were reported in systemic lupus erythematosus disease (SLE) [4]. The higher levels of *cf*DNA were reported in 1977 when the radioimmunoassay technique was used to compare the quantities of *cf*DNA of cancer patients and normal persons [5]. In 1989, the presence of *cf*DNA was detected in the plasma of cancer patients. After that, the presence of *cf*DNA was proven through various studies; scientists discovered that this DNA has unique mutations and epigenetic alternations. In the 1990s, the Human Genome Project demonstrated that *cf*DNA has tumor-specific mutations. Most of the cancer research was focused on *cf*DNA as compared to *cf*RNA because of its stability and detectability; however, numerous studies have speculated that *cf*RNA may have more potential compared to *cf*DNA [6,7,8].

Along with multiple types of *cf*DNA and *cf*RNA, various other entities are released by tumor cells, including circulating tumor cells (CTCs) and tissue DNA (tDNA). The existence of CTCs has been known since 1869 as these are commonly present in cancer patients. In 2007, CTCs were enlisted as biomarkers for cancer diagnoses; multiple kits are available on the market. However, CTCs are heterogenous seeds of cancer, and one in a million CTCs have a chance of developing cancer. CTCs are diagnosed by digital PCR, RNA sequencing, next generation sequencing (NGS), mass cytometry, and CellSearch; nonetheless, rare and low frequencies of tumor-causing CTCs make them difficult options for diagnoses [9]. Detection of tissue DNA (tDNA) was an attractive option a few years ago; however, its invasive nature, low frequency, tissue heterogeneity, and inaccuracy due to sampling locations resulted in scientists moving away from this option [10,11]; scientists have since developed noninvasive liquid biopsies, which detect cell-free DNA in blood and plasma [12]. There are three types of cell-free DNA, as explained below.

⯀ *Circulating* tumor DNA
⯀ *Cell-Free* fetal DNA
⯀ *Cell-Free* mitochondrial DNA

All types of *cf*DNA have their importance and applications which are explained in Table 1. The unique genetic makeup of *cf*DNA has given it multiple applications in diagnoses and therapeutics. For example, fetal *cf*DNA was discovered in 1997 in maternal circulation; since then, it has been used to monitor the health, gender, and genetic disorder of the fetus, especially Down’s syndrome [13]. Furthermore, mitochondrial *cf*DNA has application in diagnosing cardiovascular diseases [14], diabetes, acute myocardial infarction, cancer, and internal body trauma [15]. Finally, tumor *cf*DNA is an important diagnostic marker for cancer [16] and SLA diagnosis. This paper focuses on cell-free tumor DNA and *cf*RNA as diagnostic and prognostic markers for different types of cancer.

In this review paper, we first discuss and summarize the secretion mechanisms and characteristics of *cf*DNA and *cf*RNA. Afterward, both are discussed as diagnostic and prognostic biomarkers for cancer. In addition, their applications in minimal residual disease detection are explained in detail. We believe that a thorough understanding of the size profiles, quantitative measurements, and analyses of genetic repeats in *cf*DNA and *cf*RNA could assure reliable results in clinical practice for cancer diagnoses and therapeutics. At the end of the paper, the roles of *cf*DNA and *cf*RNA in cancer therapeutics are discussed.

## 2. Secretion

The biological and molecular analyses of cancer cells reveal the release of intracell entities, such as proteins, vesicles, nucleic acids, and many others from cancer cells. Among these entities, *cf*DNA and *cf*RNA are the most notable due to their potential to improve cancer management. These are secreted by multiple methods using active and dead cells by using various pathways, which are explained here.

### 2.1. Secretion of cfDNA

Although *cf*DNA was discovered more than 80 years ago, its molecular origins and secretion mechanisms are poorly understood. Several pathways and sources have been reported in the literature to explain its origin. A few major sources of *cf*DNA in the blood include apoptosis, necrosis, NETosis, and extracellular vesicles. The details of the secretion of *cf*DNA are reviewed in this paper. Apoptosis was the first source of *cf*DNA that was reported by Wyllie et al. [20] and similarities were found between *cf*DNA and DNA extracted from apoptotic cells leading to the conclusion that there was indeed a connection between apoptosis and *cf*DNA. During apoptosis, the DNA molecules were usually cleaved into 50−300 kb size fragments, which were further degraded into 167 bp long nucleosome units. Furthermore, Stroun et al. reported that lymphocytes and cultured cell lines, including HL-60, spontaneously release a nucleoprotein complex within a homeostatic system and preferentially release newly-synthesized DNA [21].

Necrosis is another source of *cf*DNA in the blood and generates larger fragments of *cf*DNA. The size of *cf*DNA from the necrotic origin is typically ~10k bp [22]. Therefore, necrotic *cf*DNA is not seen as commonly as apoptotic *cf*DNA in the bloodstream [23]. The post-translational modifications are responsible for histone citrullination in NETosis, which causes chromatin de-condensation and cell death. Evidence of a relationship between *cf*DNA and NETosis has been found in many studies [24,25]. In one study, increased levels of *cf*DNA were found in septic patients and indicated a correlation of NETosis with *cf*DNA secretion [26]. Another important source of *cf*DNA is extracellular vehicles (EVs); several studies have demonstrated that the uptake of RNA and DNA is done specifically as genetic material, with specific sequences and properties only being found inside EVs. The apoptotic EVs are formed due to programmed cell death and membrane blebbing and are then released from the apoptotic bodies containing fragments of degraded DNA, including *cfDNA* [27]. The stability of DNA is increased by being packaged inside EVs, resulting in protection from the external environment and nucleases. It also prevents recognition by the immune system. Cancer cells generate tumor EVs, which carry mtDNA, tumor DNA, and other mutated genetic material [28]. These cells tell the status of genetic mutation and the level of amplification of oncogenes, i.e., c-Myc [29]. These EVs are responsible for the transfer of encapsulated DNA to the target fibroblasts. Each type of EV encapsulates DNA with a specific type of mutation; therefore, *cf*DNA secreted from EVs can be used as a potential biomarker for cancer diagnosis [30].

**Figure 1:**
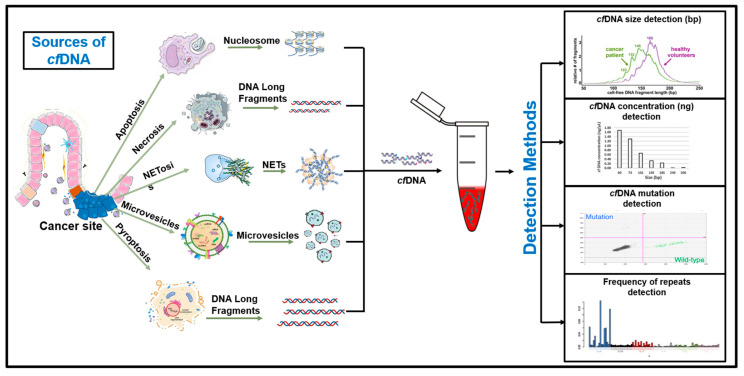
Secretion of cfDNA from tumor cells. Cell death by apoptosis, pyroptosis, or necrosis is one of the most important sources of ctDNA in body fluids; however, even without cell death, cfDNA/ctDNA has been found in the medium. This means that live cells can actively release cfDNA. Due to autophagy and exosome activity, the active secretion of cfDNA through microvesicles has also been observed. Once the cfDNA is released into the body fluids, it is detected by various methods, based on its size, concentration, frequency of repeats, or presence of mutations.

Lastly, pyroptosis (caspase 1-dependent cell death) is an important source of *cf*DNA, which occurs as a result of multiple stimuli, such as caspase activation, an immune-inflammatory reaction, or microbial stimulus [31]. Upon stimulation, pores that are 2.5 > micrometer size are formed, causing the inward flux of water and ions resulting in cell swelling and larger sizes. Proinflammatory substances, such as IL-1β and IL-18, are released into the bloodstream, triggering inflammatory and autoimmune responses. One study demonstrated that chronic inflammation activation helped prevent tumors and cancer [32]. The secretion mechanism of *cf*DNA is explained in Figure 1. The *cfDNA* was released along with other components of the cell after the membrane pore formation. Membrane lysis is not required in pyroptosis; instead, pores are enough to transport the material into the cell surroundings [33].

### 2.2. Secretion of cfRNA

**Figure 2:**
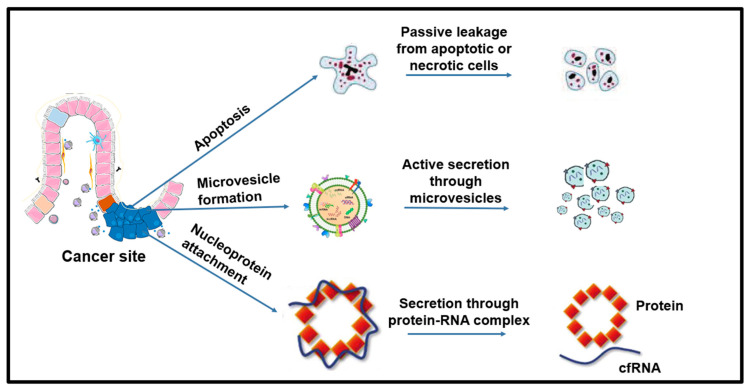
Sources of cfRNA. Similar to ctDNA, it is secreted into the body fluids through cell death events or by attaching itself to the nuclear proteins. The presence of cfRNA plasma can reflect the phenotypic alterations of localized sites of cancer as well as a systemic host response.

The presence of *cf*RNA has been confirmed in various body fluids, including blood, plasma, serum, and saliva. There are three main sources of *cf*RNA in the blood, as described in Figure 2.

⯀ Passive leakage from the tumor or apoptotic or necrotic cells;
⯀ Active secretion through microvesicles;
⯀ Secretion through nucleoproteins or protein-RNA complex.

The *cf*RNA is relatively unstable and easily degrades; therefore, it has to be packaged inside other molecular entities for their transport. Predominantly, extracellular membrane vesicles, exosomes, and microvesicles are used as transport vehicles; furthermore, nucleoproteins bind *cf*RNA and carry it outside the cell. The use of a vehicle depends on the origin of the *cf*RNA. For example, after programmed cell death, *cf*RNA is encapsulated inside extracellular membrane vesicles (EMVs) used as the delivery system [34]. The microvesicles are excellent cellular garbage disposals that transport bioactive cellular components from the cell. Their cargo also contains *cf*RNA along with other mRNAs, non-coding RNA, and other cellular entities [35]. Lastly, the attachment of *cf*RNA with nucleoproteins or high-density lipoproteins ensures the secretion into the bloodstream without any damage or degradation.

## 3. Discerning *cf*DNA and *cf*DRNA Molecular Alterations in Clinical Management

Different studies have proven that the genetic makeups of *cf*DNA and *cf*RNA of a healthy person and a cancer patient are different due to multiple mutations resulting in different genetic makeups, copy numbers, or repetitions, making them suitable candidates for diagnoses and therapeutics. Multiple techniques, including digital PCR, NGS, and genome-wide sequencing, are used to detect the molecular alterations in the *cf*DNA and *cf*RNA of cancer patients. These molecular alterations are mostly somatic, and several studies have proven the presence of mutations in RAS, Wnt, Hippo, Nrf2, TGFβ, PI3K, Notch, and P53 genes. C.H. Brian et al. studied 73 B-cell lymphoma and follicular lymphoma patients and observed lymphomagenesis. The study proved that both B-cell lymphoma and follicular lymphoma patients have different profiles of 5-hydroxymethylcytosines of *cf*DNA, which can be used to diagnose cancer. This study proposed using 5hmC-Seal as a detection tool, as it is an ultrasensitive method requiring only a 5 mL blood sample and nanograms of *cf*DNA [36]. M. Schwaederle et al. used the NGS method to detect the molecular alterations in the *ct*DNA of 670 patients. The results demonstrated that at least 63% of patients had one mutation. The most common mutation was present in *TP53,* which was found in 33% of patients. Other common mutations were found in *EGFR*, *KRAS*, and *PIK3CA*. The authors demonstrated the potential utility and feasibility of *ct*DNA in precision medicine for cancer treatment [37].

Through multiple studies, it has been established that *ct*DNA and *cf*RNA harbor multiple genomic alterations specific to the original tumor cells. Many commercially available kits detect these specific genomic alterations and mutations, i.e., the Cobas EGFR Mutation Test v2 detects mutations in EGFR. Cancer, a heterogeneous and diverse disease, tends to develop subclonal mutations, leading to tumor resistance development. A myriad of studies has found that mutations in KRAS, RAS, and EGFR T790M, and the amplification of the MET protooncogene, give rise to resistance against anti-EGFR therapy. Such mutations also alter the expressions of several oncogenic genes, such as KRAS, TP53, PKC epsilon, Akt, and several others [38,39]. These mutations are present in *ct*DNA and can be utilized to monitor the emergence of therapy resistance. In a clinical trial, Safe-SeqS was used to monitor the therapy resistance in the *ct*DNA of 42 patients after receiving the treatment and identified the emergence of KRAS mutations in 23 patients, which led to the conclusion that rather than immediately preceding surgery, neoadjuvant therapy strategies should be used as the first treatment step in cancer management.

In the case of *cf*RNA, the quantity is increased by 20Xin a cancer patient as compared to a healthy person. In patients with lung cancer, PD-L1 levels are used to monitor the response of therapy on the patients [40]. In addition, molecular expressions of RNA were used for the pathological staging and measurement of recurrence of colon cancer [41].

**Figure 3:**
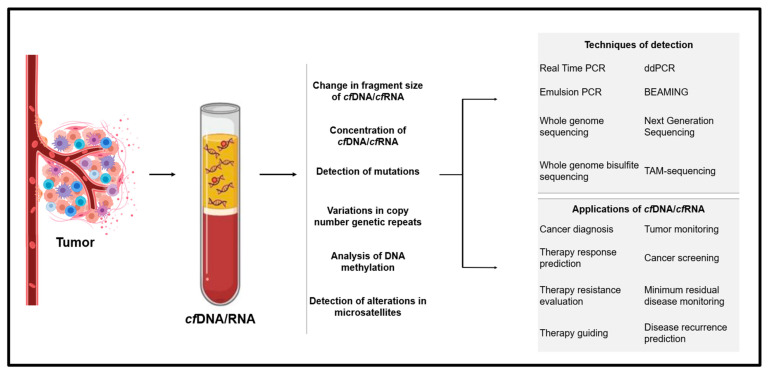
For cell-free DNA or RNA screening, the blood sample is taken from the patient and analyzed through techniques, such as qPCR, NGS WGS, etc. The non-invasiveness of a liquid biopsy makes it suitable for a myriad of applications, including cancer diagnoses, tumor burden analyses, and therapeutic analyses.

Overall, *cf*DNA and *cf*RNA have shown molecular alterations in their genetic makeups, which scientists can capture and utilize for diagnosis and treatment purposes. The details of molecular alterations, techniques that could detect them, and their applications are explained in Figure 3.

## 4. Applications of *cf*DNA and *cf*RNA

Due to the lethal (and high) cancer mortality rates, scientists have been encouraged to discover new and efficient methods for cancer detection and therapeutics. Tissue biopsies were the preferred method for cancer detection; however, they involve invasive procedures and some cancerous sites are (sometimes) unable to be reached. Therefore, scientists have identified and verified *cf*DNA, *cf*RNA, and *ct*DNA as diagnostic markers for cancer. Furthermore, due to their molecular heterogeneities, they have various clinical applications as they are used as prognostic markers, measure residual disease, evaluate the treatment responses, and identify molecular alterations. The details of various applications of *cf*DNA and *cf*RNA are explained here.

### 4.1. Role of cfDNA and cfRNA in Cancer Diagnosis

Due to the strong responsiveness and extreme dynamic properties of *cf*DNA and *cf*RNA, they are considered excellent predictors and indicators of tumor cells and DNA damage; hence, both have the potential to be used in cancer diagnostics at the commercial level.

#### 4.1.1. Role of *cf*DNA

*cf*DNA was discovered several decades ago; however, significant progress was observed after introducing NGS. In 1994, the presence of N-Ras mutations in the DNA of acute myelogenous leukemia patients [42] and K-ras sequences in pancreatic adenocarcinoma patients was reported [43]. These studies proved the elevated levels of mutations in DNA sequences of plasma, proving the presence of *ct*DNA. The concentration of *ct*DNA is negligible in an average person—it cannot be detected using typical sequencing methods; however, a cancer patient has a higher concentration, making it a potential noninvasive approach for cancer detection.

Not surprisingly, there is significant interest in the potential utility of *ct*DNA for the early noninvasive detection of cancer, with over USD 1 billion invested in companies developing such technologies in 2017 alone. Advanced PCR techniques, such as the amplification refractory mutation system (ARMS)-PCR, BEAMing (Beads, Emulsions, Amplification, and Magnetics), or droplet digital PCR (ddPCR) are utilized to identify *ct*DNA from the pool of normal *cf*DNA. The diagnostics of *ct*DNA are based on size, length, the presence of repeats, and mutations in a normal sequence. Advanced PCR techniques, such as an amplification refractory mutation system (ARMS)-PCR, BEAMing (Beads, Emulsions, Amplification, and Magnetics), or droplet digital PCR (ddPCR) are utilized to identify *ct*DNA from a pool of normal *cf*DNA. The diagnostics of *ct*DNA are based on size, length, the presence of repeats, and mutations in a normal sequence.

In the literature, the diagnoses of different types of cancer have been reported. In this regard, Panagopoulou et al. provided clinical evidence on the use of *cf*DNA as a diagnostic and prognostic marker for metastatic breast cancer [44]. In addition, Klein et al. and Rossi et al. corroborated the use of *ct*DNA as a potential prognostic and diagnostic marker for breast cancer [45,46,47,48]. Rossi et al. utilized the Guardant360 kit to analyze the *cf*DNA and Kaplan–Meier curves for the survival analysis of 90 patients. The results indicated that the average *ct*DNA fraction was 4.5% and commonly mutated genes were TP53, PIK3CA, and ERBB2. Additionally, in the baseline sample, the Fisher exact test found a strong association between the number of alterations and *cf*DNA percentage. In another study, Braicu et al. proposed *ct*DNA as a promising tool for the detection of ovarian cancer by providing clinical insights [49]. Moreover, the effectiveness and sensitivity of *cf*RNA for the detection of ovarian cancer were reported by Hannan et al. and Hulstaert et al. [50,51]. Powles et al. suggested the use of *cf*DNA with adjuvant atezolizumab to guide the immunotherapy in urothelial carcinoma patients [52]. Through various studies, clinical evidence of *cf*DNA as a potential diagnostic marker has been reported for lung cancer [53,54], pancreatic cancer [55,56], melanoma [57], B-cell lymphoma [58], and colorectal cancer [59,60,61]. Rizzo et al. reported the use of *cfDNA* for the diagnosis of biliary tract cancer [62]. Due to the presence of clinical evidence, *cf*DNA may be a potential biomarker for the detection of early–advanced-stage cancer. Different detection techniques are explained in Table 2. 

#### 4.1.2. Role of *cf*RNA

Elevated levels of *cf*RNA have been found in the blood of cancer patients; it can potentially be used as a non-invasive biomarker of cancer detection [63]. In 1999, Lo et al. reported on the elevated levels of *cf*RNA in nasopharyngeal carcinoma patients (~105 to 106 copies per cell) and suggested that it may be used as a diagnostic marker for nasopharyngeal carcinoma [64]. Cancer patients can have organ-specific transcripts of RNA, which could be detected to confirm the presence of cancer. RNA sequencing is another popular method that is used to study and research RNA. In a study, Larson et al. used transcriptome-wide characterization of *cf*RNA to identify breast cancer; they studied 57,820 genes and compared them with healthy humans. The results showed that 68% of genes present in cancer patients were different from healthy humans [65], which may be used for cancer diagnosis. Recently, Ring et al. used an epitope-independent approach for the isolation of whole transcriptome RNA-Seq of circulating tumor cells and proposed it to be used in the biopsies of macrometastases [47]. The genes associated with cancer were highly expressive in tumor cells indicating that the analysis of *cf*RNA can help in cancer discovery and understanding.

Various studies have advocated for the presence of mutations and different genetic information in the *cf*RNA of cancer patients when compared to healthy patients. Hieter et al. performed plasma *cf*RNA-sequencing to use *cf*RNA as a biomarker for hepatocellular carcinoma and multiple myeloma, showing that *cf*RNA has different expressions throughout the cancer progression stages; therefore, it is possible to identify the precancerous and cancerous conditions of patients through the *cf*RNA-based analysis [66]. The diagnosis of cancer through *cf*RNA can also be conducted by using a urine analysis; Kim et al. reported that the urine of bladder cancer patients has higher levels of *cf*RNA compared to healthy persons. Multiple studies have shown the effective diagnostic capabilities of *cf*RNA in different cancer types, including breast cancer [67], colorectal cancer [68], lung cancer [69], and many others.

**Table 2:** Techniques for the detection of ctDNA and cfRNA.

Sr number	Technique Name	Description	Sensitivity (Lower Limit of Detection)	Specificity	Limitations	Cancer Type
1	Quantitative polymerase chain reaction	Amplifies the genes in real-time	0.01–0.1%	90% for breast cancer [70]	Needs standard, prone to errors, primer design depends on results	Non-small cell lung cancer, breast cancer
2	Droplet digital polymerase chain reaction	Water–oil emulsion droplet technology-based PCR	0.01–0.1%	100% [71]	Lower quantification, loss of linearity at a high concentration	Lung adenocarcinomas, squamous cell carcinoma, neck cancer, breast cancer, gastric cancer, and others
3	Beads, emulsion, amplification, and magnetics	Combination of emulsion PCR and flow cytometry ultrasensitive technique	<0.1%	----	Single mutation per test; lacks standard data	Blood cancer, colorectal cancer
4	Cancer personalized profiling by deep sequencing	NGS-based method for ctDNA detection	0.01%–2.0%	96% [72]	For selected alterations across targeted regions	Cervical squamous cancer, bladder cancer, esophageal, lung cancer
5	Whole-genome sequencing	Analysis of the whole genome	2 × 10^−3^ [73], up to 10^−5^ shown by different studies [74].	98.4% for somatic SNV and indels [75]	Expensive, comparatively low sensitivity and specificity, large amounts of data	Gastric cancer, pancreatic cancer, breast cancer
6	TAm-Sequencing	Identify low-frequency mutations in *ct*DNA	2%	97% for ovarian cancer [76]	Less comprehensive	Breast cancer, hepatocellular carcinoma
7	Whole exome sequencing	A sequencing-based technique to study protein-coding regions in the genome	Generally 5% [77], some studies show 50% [78]	99.9% [79]	Rare variants affect the sensitivity, restricted only to exon regions	Metastatic melanoma, multiple myeloma
8	Whole-genome bisulfite sequencing	NGS-based technique to find out the methylation status of cytosine	---	99% [80]	It is difficult to differentiate between substitutions and epiallele changes; single reference genomes are not enough to discriminate the changes. It is expensive and generates a large amount of data [81]	Breast cancer, prostate cancer

#### 4.1.3. Challenges in cfDNA and cfRNA Diagnoses

Due to the small quantities of both *ct*DNA and *cf*RNA, early cancer detection is filled with cautionary tales that highlight the challenges. For example, one study [82] proposed that the fundamental limitations for such a ctDNA-based early detection test, beyond the current state-of-the-art, require around 100x more sequencing bandwidth and improved variant interpretation. Subsequently, Phallen et al., in the most comprehensive study of early-stage cancers to date, reported the detection of somatic alterations in 50–75% of patients depending on histology [83].

A positive relationship between tumor burden and *cf*DNA has been found by Valpione et al. and Xu et al. [84,85]; however, a few studies suggested otherwise [86]. In the case of *cf*RNA, only a few nanograms are available for detection, which may not be enough for most detection techniques. *cf*DNA and *cf*RNA represent complex mechanisms of cancer biology and their relationship with tumor burden have yet to be fully explored, which is a challenge for cancer diagnosis.

False negatives and false positives are major challenges that arise during *ct*DNA detection. The major reasons for false negative results are the low signal-to-noise ratio, tumor destruction, and short half-life of *ct*DNA. A low signal-to-noise ratio is an important challenge that arises in *ct*DNA detection, especially in early cancer detection. When cancer is benign or just starting to metastasize, *ct*DNA represents a tiny percentage of cell-free DNA, perhaps less than 0.01% of a 5 mL sample of plasma. At such low rates, *ct*DNA detections will need to improve significantly and be specialized to reduce the chance of error. A similar situation is true for *cf*RNA because blood contains mRNA, miRNA, and RNA fragments, which act as noise and contribute to the false detection of RNA [69]. In addition, the shorter half-life of *ct*DNA and *cf*RNA is another challenge that causes a range of problems, from tracking tumor heterogeneity to precision treatment.

Clonal hematopoiesis is one of the factors that negatively affect *ct*DNA detection. In this process, somatic mutations in hematopoietic stem cells accumulate, which disguise themselves as tumor mutations and create biological noise in cancer detection. In this regard, Liu et al. and Razavi et al. provided clinical evidence of the prevalence of clonal hematopoiesis mutations in *cf*DNA, which can affect the detection of tumor mutations [87,88]. To avoid this noise, different models of high coverage depths and ultralow error rates for NGS have been presented; however, for now, these are not accessible due to their high costs and difficult approaches.

### 4.2. cfDNA and cfRNA Roles as Prognostic Markers

In recent years, anticancer therapies have explored tumor biology-driven therapeutics, mainly focusing on prognostic and predictive biomarkers of cancer to improve cancer therapeutics. The prognostic biomarkers objectively evaluate the future outcomes of the treatment plans for specific patients and the chances of cancer reoccurrence. This helps select the cancer patients that can benefit from specific treatment plans. Both *cf*DNA and *cf*RNA are considered prognostic markers for various cancer types.

#### 4.2.1. cfDNA

Fernandez-Garcia et al. compared the conventional biomarkers for breast cancer with *ct*DNA and *cf*DNA for their potential as prognostic markers. They compared the levels of *ct*DNA and *cf*DNA of 194 patients with CA15-3 and alkaline phosphatase values. The results indicated that both *ct*DNA and *cf*DNA are good predictors of the overall response of survival; however, only *cf*DNA is the predictor of progression-free survival [89]. This study proved that both *ct*DNA and *cf*DNA are good prognostic markers of metastatic breast cancer. Liu et al. performed a meta-analysis to understand the prognostic relationship between prostate cancer and *cf*DNA. This study proved that the higher concentrations of *cf*DNA are related to progression-free survival and the overall survival of prostate cancer [90]. Apart from prostate cancer, *cf*DNA has been proved to be a potential prognostic marker for colorectal cancer. Basnet et al. reported the association between higher concentrations of *cf*DNA and recurrence-free survival and overall survival of colorectal cancer [91]. A few studies have explained the role of *cf*DNA in ovarian cancer, such as the one by No et al., who conducted the mutational analysis for the RAS oncogene family, beta-2-microglobulin, the ATP-binding cassette subfamily F member 2 and claudin 4, and levels of *cf*DNA. In this study, *cf*DNA levels from B2M and CA125 or CA19-9 found no relationship with cancer prognosis. However, the *cf*DNA of RAB25 had variations in the copy number, which could be used as a prognostic marker for ovarian cancer [92]. Bortolin et al. also demonstrated the relationship between *cf*DNA levels and lung cancer in 22 patients, proving that *cf*DNA could be used as a prognostic marker for high-risk NSCLC cancer patients [93]. These studies have proven that tracking *ct*DNA can help predict the effectiveness of therapy and the recurrence of cancer in patients.

#### 4.2.2. cfRNA

Many studies have reported that *cf*RNA has organ-specific transcripts, which can undergo temporal changes due to the development of cancer and tumors [65,94,95]. *cf*RNA not only predicts cancer and tumors but also pregnancy deliveries and preterm births [96]. In addition, it can distinguish among cancer stages and types. In a recent study, Hieter et al. distinguished the cancers from their premalignant conditions using *cf*RNA profiles and predicted the occurrence of cancer [66]. Raez et al. analyzed and measured *cf*RNA expression to monitor the clinical responses of patients with non-small cell lung cancer and reported that *cf*RNA can be used as a predictive tool for cancer onset and progression. In another study, Pucciarelli et al. used *cf*RNA to monitor the tumor response of patients with rectal cancer while they were receiving chemotherapy; the results indicated that *cf*RNA along with telomere-specific reverse transcriptase mRNA can be used to predict and measure the effects of chemotherapy [97].

Although many studies mention that *cf*RNA may be an excellent tool for cancer prediction [61,98], it has yet to be fully explored. Moreover, other than *cf*RNA, other types of RNA, including miRNA, mRNA, and circular RNA, are found to be excellent predictors; several studies have proven their prognostic potential [99,100].

### 4.3. Role of cfDNA and cfRNA in Measuring Residual Disease

After cancer treatment, a small amount of cancer cells remains inside the body, resulting in a relapse of the disease, as these are capable of activation and proliferation after a few months or years. In general, in clinical practice, flow cytometry, PCR, and NGS are used for the detection. However, scientists have found that *cf*DNA and *cf*RNA can detect minimal residual disease (MRD). The clinical applications of liquid biopsy are described in Table 3. 

#### 4.3.1. ctDNA

A. A. Chaudhuri et al. analyzed the *ct*DNA samples of 40 patients (treated with curative-intent first-line therapies) and 54 healthy persons through PET-Scan and CAPP-seq techniques by targeting 128 genes. The dates pre- and post-treatment for lung cancer were compared. The results demonstrated that the patients in which *ct*DNA was detected had the recurrence of cancer. This study proved that *ct*DNA could potentially detect MRD and predict the relapse of the disease in patients [73]. C. Abbosh et al. provided a phylogenetic analysis of *ct*DNA, the proof of chemotherapy resistance, and the chance of lung cancer recurrence [101]. Leal et al. compared the alterations of *ct*DNA with the DNA of white blood cells of the same patients and proved that *ct*DNA could predict recurrence when analyzed within nine weeks after treatment [102]. Murillas et al. analyzed the potential of *ct*DNA to detect MRD and predict the relapse of disease after the treatment of breast cancer in 55 patients. The results demonstrated that *ct*DNA could accurately predict the relapse of the disease. They also used a mutation tracking analysis in this study, which increased the sensitivity and specificity of the detection [103].

#### 4.3.2. cfRNA

*cf*RNA, similar to *ct*DNA, has the potential to measure the disease burden and MRD. Most of the studies report the capability of *ct*DNA to measure MRD; however, a few report that *cf*RNA can potentially measure MRD [104]. Rossi reported that *cf*RNA can detect MRD and the reoccurrence of cancer; its capacity and performance were compared via a flow cytometry-based analysis [105].

Detecting MRD requires highly sensitive approaches that can monitor MRD through patient-specific and mutation-specific analyses. The analysis of MRD may benefit humans as the major challenge in cancer treatment involves the recurrence of disease after completing painful treatments and surgeries. MRD is an issue that scientists have discussed and researched for decades. Currently, MRD has become a focus of research for the prediction of disease relapse. The detection of *ct*DNA after treatment can identify the patients with MRD who are at a higher risk of cancer recurrence. Multiple studies have demonstrated that *ct*DNA can be a potential diagnostic marker of MRD and predict the recurrence of the disease.

**Table 3:** Clinical applications of *ct*DNA.

Cancer Types	Clinical Management	Conclusion	References
Breast Cancer	Assessment of early *ct*DNA dynamics in 149 patients	Assessment of early dynamic changes in *ct*DNA can predict the efficacy of targeted therapy	[106]
	Prediction of recurrence of cancer	Predicted metastatic relapse with high accuracy	[103]
	Detection of PIK3CA mutations	*Ct*DNA can be used for the detection of PIK3CA mutations	[107]
Lung cancer	Cancer diagnosis and therapeutics	EGFR L858R and T790M mutations can be detected in *ct*DNA using NGS and ddPCR	[108]
	Detection of cancer at IA, IB, and IIA stages	Found an association among tumor stage, subtype, and *cf*DNA concentration, and demonstrated the utility and feasibility of targeted sequencing for *ct*DNA monitoring	[109]
	Monitoring treatment response and drug resistance	Proved the decrease of mutant copies after treatment and monitored the emergence of secondary mutations	[110]
	Detection of copy number variations in tumor cells	Copy number changed in BAX, P53, CASP3, SOX2, GRB2, SOS1, MAPK1, and a few other genes of cancer patients	[111,112]
	Diagnostic marker	The presence of *ct*DNA in cancer patients was found to be eight times higher than in healthy persons	[113]
Colon cancer	Adjuvant therapy	Provided evidence of the potential use of *ct*DNA with adjuvant novel drugs for cancer patients	[114]
Colorectal cancer	Marker for cancer recurrence	Postoperative *ct*DNA positivity may be associated with the recurrence of cancer	[115]
	Predicting response to neoadjuvant chemoradiotherapy	Combination of MRI and *ct*DNA potentially improves the predictive performance of cancer	[116]
	Novel biomarker Optimizing chemotherapy	Described that *ct*DNA can potentially be utilized for diagnosis and personalized medicine for colorectal cancer	[117]
Melanoma	Monitoring of relapse in stage III	*ct*DNA identified the highest risk stage III melanoma patients and helped in adjuvant therapy decisions	[118]
	Detection of *ct*DNA levels in patients treated with anti-PD1 therapy	The evaluation of *ct*DNA during anti-PD1 antibody therapy provided reliable information	[119]
Leiomyosarcoma	Detection of genetic variants using Guardant360	The identification of molecular alterations can help develop therapy, targeting TP53, cell cycle, and kinase signaling pathways	[120]

## 5. Clinical Trials

Scientists have shifted their interest to personalized medicines due to their high accuracy and ability to predict the recurrence of cancer. Oncologists have focused their research on *ct*DNA and *cf*DNA as biomarkers for tumor profiling. In addition, the noninvasive nature of a *cf*DNA-based analysis makes it a good treatment option for patients. As a result, several types of *cf*DNA and *ct*DNA-based cancer management systems have been developed and clinically tested.

### 5.1. ctDNA

The details of a few major clinical trials, which were multicentric, multinational, and used *ct*DNA as the detection method in larger population sizes, are given below.

#### 5.1.1. TARGET Study

Tumor characterization to guide experimental targeted therapy, known as TARGET, was a phase I study conducted at Manchester Cancer Research Center on 550 patients to determine the feasibility of using *ct*DNA to diagnose cancer, utilizing mutational analyses of advanced-stage cancers. Results were compared to the tissue-based testing. The study was divided into two parts. The first part was conducted on 100 patients to assess the feasibility of the analytical workflow and data turnaround (of two to four weeks) for clinical decision-making. Then, the clinical utility of *ct*DNA was analyzed on the remaining 450 patients who were selected based on ctDNA and/or tumor genomic profiling. The results demonstrated that *ct*DNA could detect 54 out of 69 mutations, giving a 78.3% detection rate [121]. Foundation medicine testing was also conducted on 39 patients, where *ct*DNA results were compared with the FoundationOne panel. This trial proved that *ct*DNA is a feasible option for the guidance of selecting specific treatment regimens for cancer patients. The results of the TARGET study encourage routine implementation of *ct*DNA testing as an adjunct to tumor testing.

#### 5.1.2. MONALEESA Study

The MONALEESA study was one of the largest clinical trials that studied the feasibility of using *ct*DNA as the biomarker for cancer diagnoses and therapeutics. In all three MONALEESA trials, a total of 1503 patients with advanced breast cancer were recruited for the analyses. The trials were conducted to observe the effects of the combination of ribociclib with endocrine therapy and analyses were conducted via a *ct*DNA evaluation using NGS. Due to the results, the researchers proposed the use of ribociclib with endocrine therapy as a first-line treatment for breast cancer patients. During the *ct*DNA analysis, genomic alterations in PIK3CA, TP53, ZNF703/FGFR1, and ESR1 were observed; however, irrespective of the genomic alterations, the treatment was found effective to reduce the disease progression [122].

#### 5.1.3. I-PREDICT Study

I-PREDICT is the abbreviation for Investigation of Profile-Related Evidence Determining Individualized Cancer Therapy. It involved an open-label navigational investigation and a prospective study conducted at the University of California, San Diego Moores Cancer Center, and Avera Cancer Institute South Dakota on 149 patients. The objective of this study was to assess the success of individualized cancer therapy by analyzing the molecular profiles of the patients. Multiple techniques were utilized for molecular profiling, including *ct*DNA analysis, NGS, tumor mutational burden, programmed death-ligand 1 immunohistochemistry, and microsatellite instability status. A total of 49% of these stage IV patients were treated using personalized treatments consisting of customized drug regimens. The study proved that identifying molecular alterations for larger fractions of alterations is better than targeting fewer somatic alterations because it yields high matching scores, improving disease control rates and longer survival and progression-free rates. The success rate of this trial was higher than in other precision medicine trials. There were several factors responsible for the increased success rate, including the use of sophisticated diagnostic techniques, such as *ct*DNA, and combining the results with targeted drug delivery. In conclusion, this trial proposed that the paradigm for precision oncology can be improved by using multi-drug combinations targeting multiple identified molecular alterations [123].

In conclusion, the TARGET, I-PREDICT, and MONALEESA studies nicely demonstrated the feasibility of using some of these innovative approaches in cancer precision medicine, including *ct*DNA for identification of molecular alterations and guidance of patients to clinical trials, drug combinations to target as many molecular alterations in a patient as possible, and treatment algorithms based on the RNA expressions that necessitate normal adjacent tissue samples.

Apart from these clinical trials, to date, 20 different clinical trials have been conducted to prove the safety, efficacy, and efficiency of *ct*DNA, including PADA, Solar-1, BELLE 1, 2, 3, POSEIDON, MONALEESA I, II, III, and others.

As *cf*DNA-based diagnosis is an emerging field, several trials are ongoing. The clinical trial MERMAID-I is being conducted on lung cancer patients to study the adjuvant durvalumab along with chemotherapy and minimal residual disease, studied by analyzing the *ct*DNA of patients. Similar to MERMAID-I, MERMAID-II is a phase III clinical trial that is being conducted on patients who have completed their curative intent and are now in the surveillance mode for MRD and recurrence of disease through *ct*DNA analysis. Another ongoing trial is SUMMIT, which has a large population size (25,000), to validate the feasibility and accuracy of early cancer detection by using *ct*DNA. There are many other ongoing trials related to the use of *ct*DNA for cancer diagnoses, therapeutics, and MRD; only a few are described here.

### 5.2. cfRNA

Due to the potential of *cf*RNA, scientists have focused on *ct*DNA/*cf*DNA-based studies and several clinical trials have been conducted. In the case of *cf*RNA, not much data are available and fewer clinical trials were conducted (details are given below).

#### 5.2.1. ICE-PAC

The clinical trial ICE-PAC was a phase II, multicentric, nonrandomized trial conducted in Australia between 2017 and 2019. The main objective of the trial was to analyze the effects of the PD-L1 checkpoint inhibitor avelumab in combination with stereotactic ablative body radiotherapy (SABR) in prostate cancer in 31 male patients. This trial also analyzed *cf*RNA as a biomarker and used it to measure pathogenicity. The results of this trial indicated that *cf*RNA has the potential to be used to measure the tumor response and the development/emergence of resistance [124].

#### 5.2.2. NCT02853097

This was a research trial that was conducted to analyze the correlation of *cf*RNA with the progression of prostate cancer. For this purpose, the expressions of PD-L1, TIM-3, PCA3, AR, and AR-V7 were measured with the help of *cf*RNA, and the results were compared to healthy individuals. The results indicated that PCA3 was not detected in healthy humans; however, AR, AR-V, and TIM-3 were found in 41%, 7%, and 43% of cancer patients, respectively. The study concluded that *cf*RNA may be potentially used in the early diagnosis of cancer and reoccurrence of cancer after treatment [125].

Many clinical trials on *cf*DNA and *ct*DNA have proven their efficiency and utility; the FDA has even approved some of the kits. The literature has shown that weak and highly variable signal detections make *cf*RNA difficult to measure and analyze. Many research groups are attempting to update their protocols for the robust and reproducible detection of *cf*RNA, such as the nCounter platform [126]; we hope that we will have more results to publish and products to review in the future.

## 6. Food and Drug Administration Approved *ct*DNA and *cf*RNA Tests

For decades, scientists have attempted to develop noninvasive diagnostic and treatment methods for cancer patients. Due to the non-invasiveness, effectiveness, and sensitivity of *ct*DNA, many assays and detection kits have been developed. Many are still in the trial phase, and a few have been FDA-approved.

Cobas^®^ EGFR Mutation Test v2 (Roche, Basel, Switzerland) was one of the earliest tests approved by the FDA (on 1 June 2016) to detect cancer, using blood. It is recommended for non-small cell lung cancer and identifies mutations in EGFR, including exon 19 deletions or exon 21 substitutions.

Signatera (Natera Inc., Austin, TX, USA) is a *ct*DNA-based cancer diagnostic assay that has been proven to be a suitable candidate for predicting the long-term outcomes of surgery and anticancer treatments.

Guardant360^®^ (Guardant Health, Lansdale, PA, USA) is another FDA-approved liquid biopsy (7 August 2020) that has been tested on more than 150,000 patients and approved by 7000 oncologists. It is recommended for advanced cancer patients and is used as a companion diagnostic test for lung cancer patients who have EGFR mutations. In addition to Medicare, it is also covered by some private players. FDA approval of this test was an important milestone in the history of oncology. Currently, this test is recommended for colorectal, breast, lung, and prostate cancer.

FoundationOne CDx (Foundation Medicine, Cambridge, MA, USA) is one of the blood tests approved by the FDA (on 26 August 2020); it is used to detect tumors in cancer patients. It can detect the mutations of 300 genes in more than 30 cancer types. In addition to tumor profiling, it is used as a companion diagnostic test. It has been approved for solid cancers, including lung and prostate cancer. Apart from the mutation analysis, it also provides information about tumor burden and microsatellite instability.

Other than the assays mentioned above, several other kits are available commercially to detect *ct*DNA but are not FDA-approved. These kits include QIAamp Circulating NucleicAcid kit, the Maxwell^®^ RSC ccfDNA Plasma kit, NucleoSpin Plasma XS, MagMAX™ Total Nucleic Acid Isolation kit, and the Chemagic Circulating NA kit. In the case of *cf*RNA, only one assay is approved, known as the PROGENSA PCA3 assay. It is an in-vitro test that amplifies nucleic acids and measures a specific marker of prostate cancer called PCA3 RNA. The result of this assay generates a score that correlates with the likelihood of a positive repeat biopsy. This test is suitable to analyze at-risk patient populations [127].

## 7. Other Techniques for Cancer Detection

The PET/CT technique is widely used for the detection and screening of cancer; however, it is associated with some challenges, i.e., inaccuracy, expensiveness, and radiation exposure. Currently, it is combined with other modalities to improve the issues related to accuracy. Sasamori et al. combined the PEC/CT scan with whole-body diffusion-weighted imaging and improved accuracy from 48% to 55% [128]. Moving a step forward, Lennon et al. combined *cf*DNA detection with PET/CT and reduced the chances of false positive results in *cf*DNA-based cancer diagnoses [129]. In addition, Kwee et al. and Woff et al. provided clinical evidence on the improvement of cancer detection by combining *cf*DNA with PET-based techniques [130,131]. Through these studies, it was found that *cf*DNA-based diagnosis is a promising method; however, it has yet to be fully developed, and by combining it with other techniques, the sensitivity and specificity can be improved. GeneCT is a deep learning, RNA-based classifier for the prediction of the status, stage, and origins of cancer. This system has shown good results, is expected to be further developed and may be used for commercial purposes [132].

## 8. Discussion

In clinical practice, tissue biopsy-based invasive methods are commonly used. Despite their accuracy, a few issues have been associated with them, shifting the focus of researchers toward non-invasive liquid biopsies. Although imaging-related techniques are available, their costs and false positive results make them nonpreferred techniques. Liquid biopsies have proven suitable for cancer management by providing early diagnoses, as well as MRD evaluation and tumor response analysis after treatment. They can improve cancer diagnosis and treatment by using minimally invasive or non-invasive means.

In addition to *cf*DNA and *cf*RNA, researchers are investigating circulating cells (e.g., circulating tumor cells, circulating hybrid cells, mRNA, miRNA, and tumor-associated macrophages). Currently, all of these methods are expensive, and their detections are challenging due to high variations in molecular genetics; nevertheless, it is expected that in the near future, other than *cf*DNA and *cf*RNA, there will be many options available for cancer management.

Although fewer studies are available for the analysis and exploration of *cf*RNA, it has great potential to be used as a biomarker for cancer diagnosis and treatment. It can not only detect cancer but also provide additional useful information, such as alterations in the molecular profiles, effects on the immune system or other systems of the body, and disease progression. Furthermore, it is stable in blood, making it easier for researchers to extract and analyze. In conclusion, *cf*DNA- and *cf*RNA-based liquid biopsies have shown promising results and will play important roles in cancer management and personalized medication, but there is still a need for further research.

## 9. Future Directions and Limitations

In the past decade, many studies have been conducted on the use of *cf*DNA as a potential biomarker for cancer diagnosis and therapeutics; however, there is still room for further research and improvements. Currently, *ct*DNA-based assays are potentially suitable for late-stage cancer diagnoses, prognoses, and disease recurrence predictions; however, early-stage cancer management is still uncertain. Secondly, *ct*DNA-based assays lack standardization; therefore, the standard should be developed to make this technique more appropriate and desirable. Furthermore, there is the issue of false negatives and false positives due to the presence of *cf*DNA in the normal cells of healthy humans; thus, the quantification techniques should be improved and updated to minimize the false predictions and diagnoses. Lastly, the cost is an essential factor that should be considered as the assays and kits available are not affordable for poorer populations.

One major challenge faced with a *cf*RNA analysis involves the absence of reference genes and unavailability of data; therefore, developing archives for reference data should be encouraged to solve it. Secondly, specific reference genes and tests are required for specific types of cancer; hence, the specificity of *cf*RNA-based tests should be increased. Non-specific detection of RNA has also been observed due to non-cancer-related factors, such as posttraumatic organ failure. Future research should focus on enhancing the specificities of these analyses and building standards/references for comparison purposes.

## 10. Conclusions

The analysis of *cf*DNA and *cf*RNA using body fluids is an attractive solution for cancer diagnosis, to solve, for example, the invasiveness, low frequency, and non-specificity of other techniques. They not only detect the tumor inside the body but can also analyze the effectiveness of anticancer therapy, evaluate the drug resistance capacity, and predict the chances of recurrence of cancer. There are limitations associated with these techniques, including relatively low sensitivity, lack of information about the origin, and low specificity; researchers are attempting to improve the sensitivities and specificities of these techniques. Multiple clinical trials and studies have been conducted, utilizing sophisticated sequencing and PCR-based techniques, resulting in a tremendous increase in the sensitivity and specificity of *ct*DNA and *cf*RNA. To the best of our knowledge, liquid biopsy-based assays and therapeutics are breakthroughs in the field of companion diagnostics and personalized medicine; however, concrete evidence is still required through clinical trials on a larger population.

## Figures and Tables

**Table 1:** Comparison of various types of cell-free DNA.

Sr number	Properties	Circulating Tumor DNA	Cell-Free Fetal DNA	Cell-Free Mitochondrial DNA
1	Strands	Single or double	Single or double	Double
2	Origin	Tumor cells	Trophoblastic cells [13]	Mitochondria or unknown [17]
3	Size	Less than 100 bp [18]	200 bp with dominate peaks at 162 bp [19]	Shorter fragment = less than 1 kb; larger fragments = 21 kb [14]
4	Applications	Early cancer detection, mutation analysis, cancer prediction, noninvasive cancer detection	Prenatal testing, genetic disease detection in the fetus	Forensic sciences, detection of the geographical distribution of genes, gene flow identification, human remain recognition, cancer detection
5	Advantages	Sensitive than other cancer detection techniques, can detect mutations better than biopsy, detect heterogeneous tumor cells, predict cancer reoccurrence	Increased chances to detect chromosomal disorders, noninvasive, no side effects	Lacks genetic ambiguities, higher copy number, a diagnostic and prognostic marker for multiple diseases
6	Disadvantages	Cannot be detected by FISH or ICC techniques, expensive, lack standardization	Increased chances of false positives or false negatives	No heterogenicity, lower discrimination power

## Data Availability

Not applicable.
